# Optimal pulse length of insonification for Piezo1 activation and intracellular calcium response

**DOI:** 10.1038/s41598-020-78553-2

**Published:** 2021-01-12

**Authors:** Defei Liao, Ming-Yen Hsiao, Gaoming Xiang, Pei Zhong

**Affiliations:** 1 Department of Mechanical Engineering and Materials Science, Duke University, Durham, NC 27708 USA; 2 Department of Physical Medicine & Rehabilitation, National Taiwan University College of Medicine, Taipei, Taiwan; 3 Department of Physical Medicine & Rehabilitation, National Taiwan University Hospital, Taipei, Taiwan

**Keywords:** Membrane biophysics, Neurology, Biomedical engineering, Mechanical engineering, Cell signalling, Cellular imaging

## Abstract

Ultrasound (US) neuromodulation, especially sonogenetics, has been demonstrated with potential applications in noninvasive and targeted treatment of various neurological disorders. Despite the growing interest, the mechanism for US neuromodulation remains elusive, and the optimal condition for eliciting a neural response with minimal adverse effect has not been identified. Here, we investigate the Piezo1 activation and intracellular calcium response elicited by acoustical streaming induced shear stress under various US exposure conditions. We find that Piezo1 activation and resultant intracellular calcium response depend critically on shear stress amplitude and pulse length of the stimulation. Under the same insonification acoustic energy, we further identify an optical pulse length that leads to maximum cell deformation, Piezo1 activation, and calcium response with minimal injury, confirmed by numerical modeling of Piezo1 channel gating dynamics. Our results provide insight into the mechanism of ultrasonic activation of Piezo1 and highlight the importance of optimizing US exposure conditions in sonogenetics applications.

## Introduction

Ultrasound (US) has recently attracted significant attention as a non-invasive modality for neuromodulation, such as in the brain and heart. US has been demonstrated to modulate excitable cells in the central^1–6^ and peripheral^7–9^ nervous systems. The unique combination of deep tissue penetration and spatial focusing capability of US has spurred increasing research activities with the hope for potential clinical treatment of Parkinson’s disease, epilepsy and depression^10^. Despite the growing interest, the specific mechanism responsible for US neuromodulation is largely unknown, and the optimal US exposure conditions for eliciting neural response with minimal adverse effects have yet been clearly identified^11, 12^.

Previous studies have examined the effects of pulsed ultrasound on neural activity using a wide range of insonification schemes^3, 4, 13, 14^. Various combinations of fundamental frequency (f_0_), pressure amplitude (PA), duty cycle (DC), pulse length (PL), pulse-repetition frequency (PRF), and treatment time (TT) have been evaluated. For example, Kim et al. found that 1 to 5 ms PL produces the strongest motor response in rats, and further speculated that increasing PL might recruit inhibitory neural circuits and thus raising the stimulation threshold^15^. Jan and colleagues reported that pulsed ultrasound at 300–1000 Hz PRF, 50% DC (corresponding to PL = 0.5 to 1.7 ms) and 200 ms TT produces the highest behavior response ratio in C. elegans, possibly due to the mechanical filtering effect associated with the viscoelastic properties of soft tissues^16^. Whilst the previous studies have attempted to determine the optimal US parameters for eliciting a specific neuroactivity, significant challenge exists in elucidating the underlying mechanism of action because of the complex biological systems involved in animal models.

To address this fundamental challenge, we investigate the effects of ultrasound on individual cells in culture, which allows us to correlate exclusively the mechanical with biological response of the cell under a broad range of insonification conditions. Specifically, we focus on Piezo1^17^, one of the few eukaryotic mechanosensitive (MS) ion channels that respond distinctly to US-induced mechanical stimulations^18–20^. Compared with other mechanosensitive ion channels that can be activated by ultrasound, such as two pore domain $${K}^{+}$$ channels^21^, voltage-gated $${Na}^{+}$$ and $${Ca}^{2+}$$ channels^22^, and transient receptor potential (TRP) channels^23, 24^, Piezo1 has diverse expression pattern in mammalian cells^25^, high specificity and sensitivity to mechanical force^19, 26^, and more intriguingly, exhibits frequency dependent-filtering effect in response to repetitive forces^27^. These unique features make Piezo1 a strong candidate for the ideal molecular target of US neuromodulation. Recently, we have developed a novel vertically deployed surface acoustic wave (VD-SAW) platform to deliver high frequency US stimulation and acoustic streaming induced shear stresses to single HEK293T cells transfected with Piezo1 while monitoring concomitantly the cell displacement, membrane poration, and Ca^2+^ signaling^28^. We found that under the same 20% DC and 60 s TT, US treatment at 100 ms PL (or 2 Hz PRF) produces much higher Piezo1 activation probability and intracellular calcium ($${[{Ca}^{2+}]}_{i})$$ response than its counterpart at 1 ms PL (or 200 Hz PRF). This preliminary observation suggests that an optimal US condition may exist, leading to the highest Piezo1 activation probability and $${[{Ca}^{2+}]}_{i}$$ response.

In this study, we employ the VD-SAW platform and further broaden the parameter space to determine the safe and effective range of US exposures for Piezo1 activation and ${[{Ca}^{2+}]}_{i}$ response in the HEK293T cells. In particular, we examined cell displacement, calcium response characteristics, membrane poration and cell detachment under shear stress produced by the VD-SAW transducer in the range of 18 to 74 dyne/cm^2^. Under the 50 dyne/cm^2^ shear stress level, we further adjusted the PL over a broad range from 0.01 ms to 1.0 s to identify the optimal insonification condition for safe and effective activation of Piezo1. Moreover, we utilized a four-state model of channel gating for Piezo1^17, 27^ with modifications to facilitate the interpretation of the PL-dependency in ${[{Ca}^{2+}]}_{i}$ response observed experimentally. Altogether, our results highlight the importance of optimizing US pulsating strategy for eliciting a safe and effective calcium response in cells with mechanosensitive ion channels.

## Results

### Application of shear stress to single cells by VD-SAW

**Figure 1:**
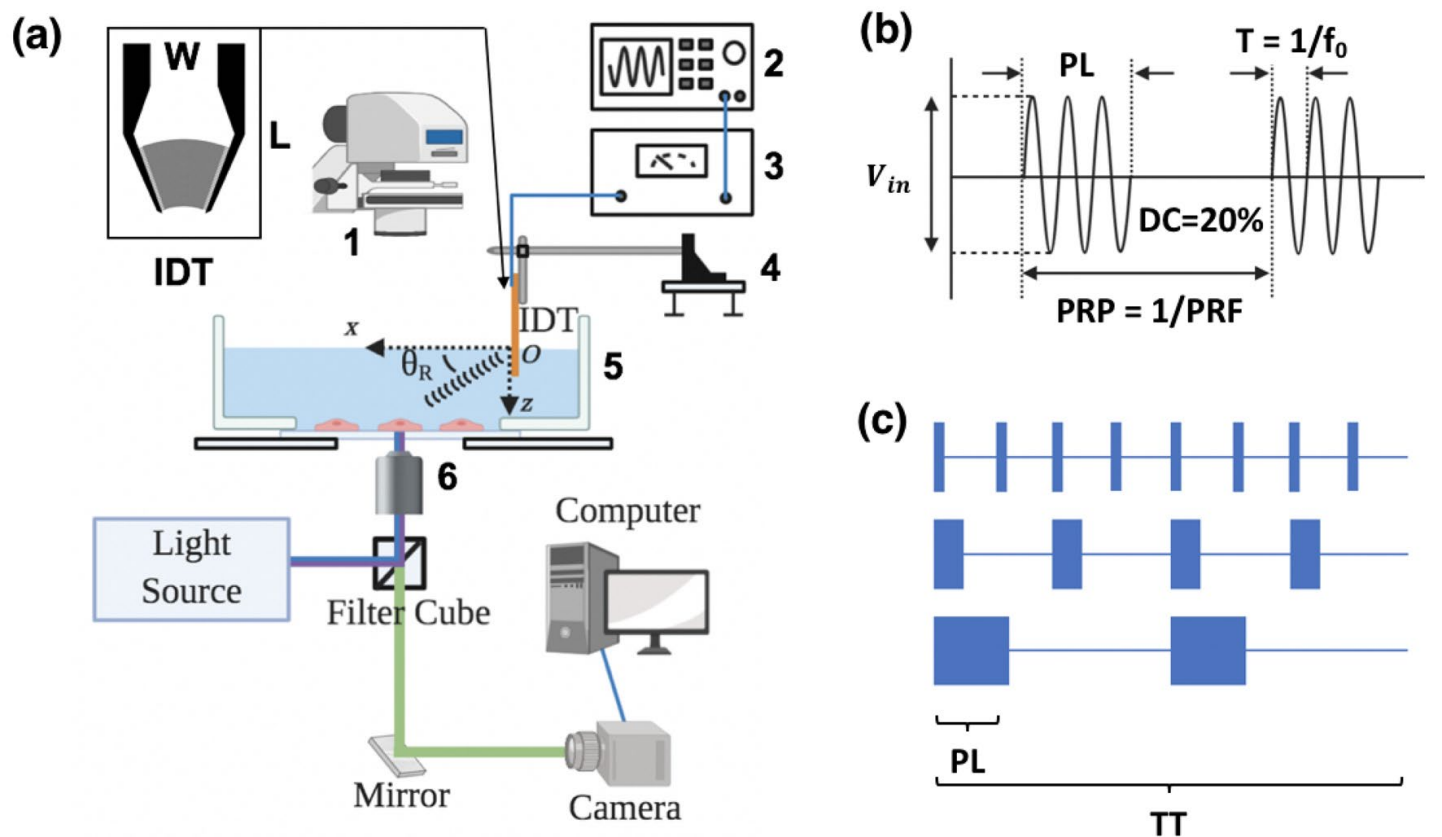
Piezo1 channel is activated by ultrasound with various insonification parameters, using our VD-SAW platform. (**a**) A schematic illustration of the combined VD-SAW transducer and fluorescent imaging system: 1: condenser, 2: function generator, 3: power amplifier, 4: three-axis micro-manipulator, 5: glass-bottom Petri dish, 6: 20 × objective lens. (**b**) An illustration of various parameters of the ultrasound pulses: PL: pulse length, T: period, f_0_: fundamental frequency, V_in_: input voltage, DC: duty cycle, PRP: pulse repetition period, PRF: pulse repetition frequency. (**c**) Diagrams depicting different insonification protocols with varying PL while maintaining a constant total ultrasound exposure energy. TT: treatment time.

We utilized a 33-MHz VD-SAW transducer to produce leaky pressure waves and apply acoustic streaming-induced shear stresses to individual HEK293T adherent cells grown on the glass-bottom of a Petri dish (Fig. 1a, see also^28^). Figure 1b illustrates the modulated electrical signals supplied to the VD-SAW transducer and associated parameters (input voltage (V_in_), period (T), TT, f_0_, pulse repetition period (PRP), PL and PRF). Figure 1c depicts our experimental protocol for varying PL while delivering a constant total insonification acoustic energy to the target cells under the same DC (20%) and TT (60 s).

**Figure 2:**
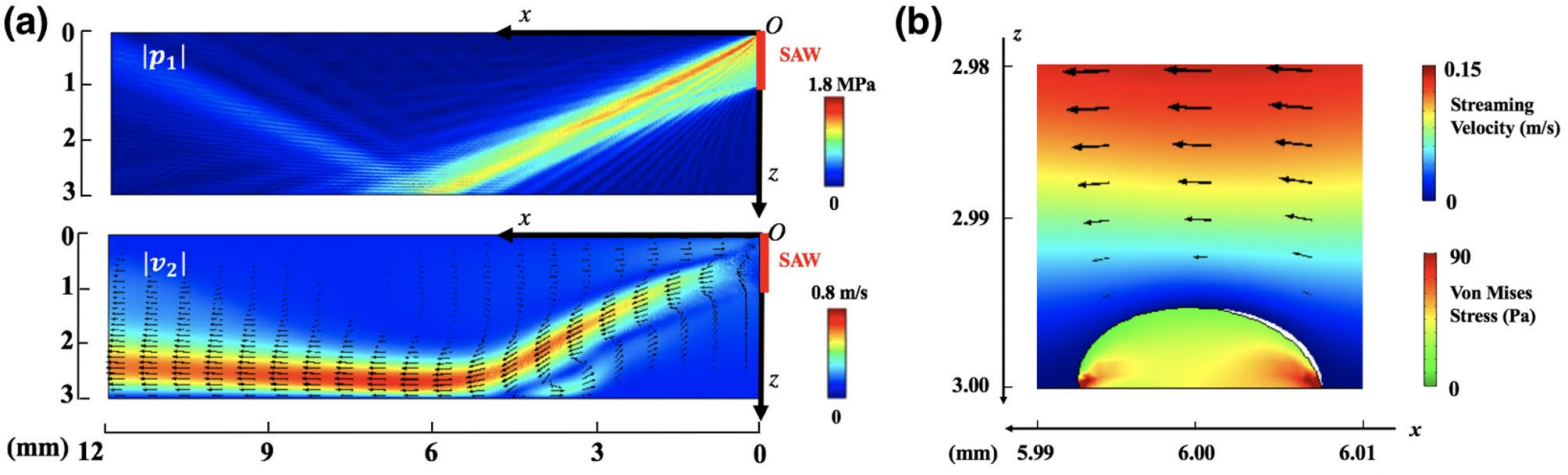
Numerical modeling reveals that the shear stress induced by acoustic streaming is the dominant mechanism for Piezo1 activation observed in the experiment. V_in_ = 80 mV (or $\uptau$ = 50 dyne/cm^2^) was used as an example. (**a**) Finite element simulation of the acoustic field produced by VD-SAW operated under CW mode. Top: the amplitude of the first-order pressure amplitude ($|{p}_{1}|$) associated with acoustic radiation force. Bottom: the amplitude of the second-order velocity ($|{v}_{2}|$) associated with acoustic streaming. (**b**) Finite element simulation shows the distribution of von Mises stress inside an adherent cell model with its center located at (x, z) = (6 mm, 3 mm) in response to streaming flow-induced shear. Black-arrow vectors indicate the direction and amplitude of the streaming velocity. White gap between the deformed cell and flow field represents the displacement of the original cell.

We performed numerical simulations by COMSOL to calculate the pressure amplitude $\left(\left|{p}_{1}\right|\right)$ and streaming velocity amplitude $(\left|{v}_{2}\right|)$ produced by the VD-SAW transducer under continuous wave (CW) mode (Fig. 2a). The vibration amplitude of the harmonic SAW measured by a custom-built optical interferometer was input as the boundary condition in the simulation (see Supplementary Fig. S1). The pressure waveforms generated by the VD-SAW in a water tank were also measured by a fiber optic probe hydrophone at z = 3 mm plane. The peak pressure amplitude (1.44 MPa) and distribution along the x-axis were found to match well with the COMSOL simulation results in a free-field (see Supplementary Fig. S2). The shear stress ($\uptau$) at the bottom of the Petri dish near the cell surface was calculated by $\uptau =\upmu \frac{d{v}_{2}}{dz}$ ($\upmu$ is the dynamic viscosity of water. At 20 °C, $\upmu =1\times {10}^{-3} {\rm{N}}\cdot {\rm{s}} \cdot {{\rm{m}}}^{-2}$). The range of shear stress produced in the target area varies from 18 to 74 dyne/cm^2^ (Supplementary Fig. S3), encompassing the previously reported values for shear-induced activation of Piezo1 in various types of cells^29–32^. In contrast, the acoustic radiation force (per unit volume) produced by the VD-SAW near the cell surface was estimated to be about 2.0 × 10^4^ N/m^3^ ^28^, which is an order of magnitude less than the threshold value (1.5 × 10^5^ N/m^3^) reported to activate Piezo1 by US^19^. Therefore, we hypothesize that acoustic streaming-induced shear stress is the dominant factor for eliciting the calcium response in this study.

Using a 2D viscoelastic model of a semi-elliptical shape with semi-major axis a = 7.5 μm and semi-minor axis b = 5 μm and associated mechanical properties relevant to HEK293T cell^33^, we simulated the response of an adherent cell to a constant shear flow to illustrate the salient features of their interaction (Fig. 2b). In particular, we observed inhomogeneous distribution of the von Mises stress inside the cell, with a high stress concentration built up at the leading edge, accompanied by a relatively small area of stress concentration produced at the trailing edge of the cell along the shear flow direction.

### Effects of shear stress on Piezo1 activation and ${[{{{C}}{{a}}}^{2+}]}_{{{i}}}$

To determine the effective and safe parameter range of VD-SAW to activate Piezo1, we simultaneously treated co-cultured P1KO (piezo1 knock-out) and P1TF (piezo1 transfected) cells within the same Petri dish by various insonification protocols. We examined the effects of shear stress on Piezo1 activation, ${[{Ca}^{2+}]}_{i}$ response, membrane poration and cell detachment. All of the cells having a normalized fluorescence (F) ratio ((F-F_0_)/F_0_) above 10% of the background fluorescence (F_0_) were considered as “responsive”; other cells that fell below this threshold were defined as “non-responsive”.

**Figure 3:**
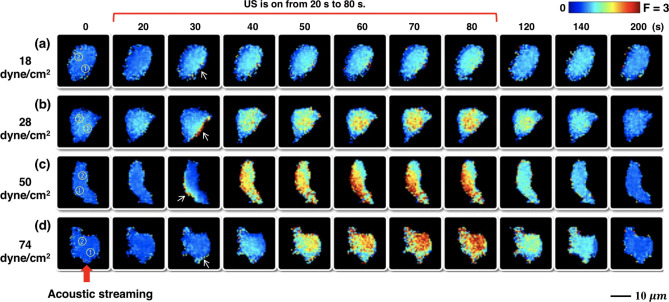
Sequential images show the temporal ${[{Ca}^{2+}]}_{i}$ ratio change in individual P1TF cells in response to four different levels of shear stress induced by acoustic streaming under 100 ms PL and 20% DC. (**a**) $\uptau$ = 18 dyne/cm^2^. (**b**) $\uptau$ = 28 dyne/cm^2^. (**c**) $\uptau$ = 50 dyne/cm^2^. (**d**)$\uptau$ = 74 dyne/cm^2^. US was on from 20 to 80 s. Circles with numbers represents the regions used for calculating *C*_*ICW*_. White arrows indicate the ${[{Ca}^{2+}]}_{i}$ response initiation site. Red arrow at the bottom indicades the direction of acoustic streaming.

**Figure 4:**
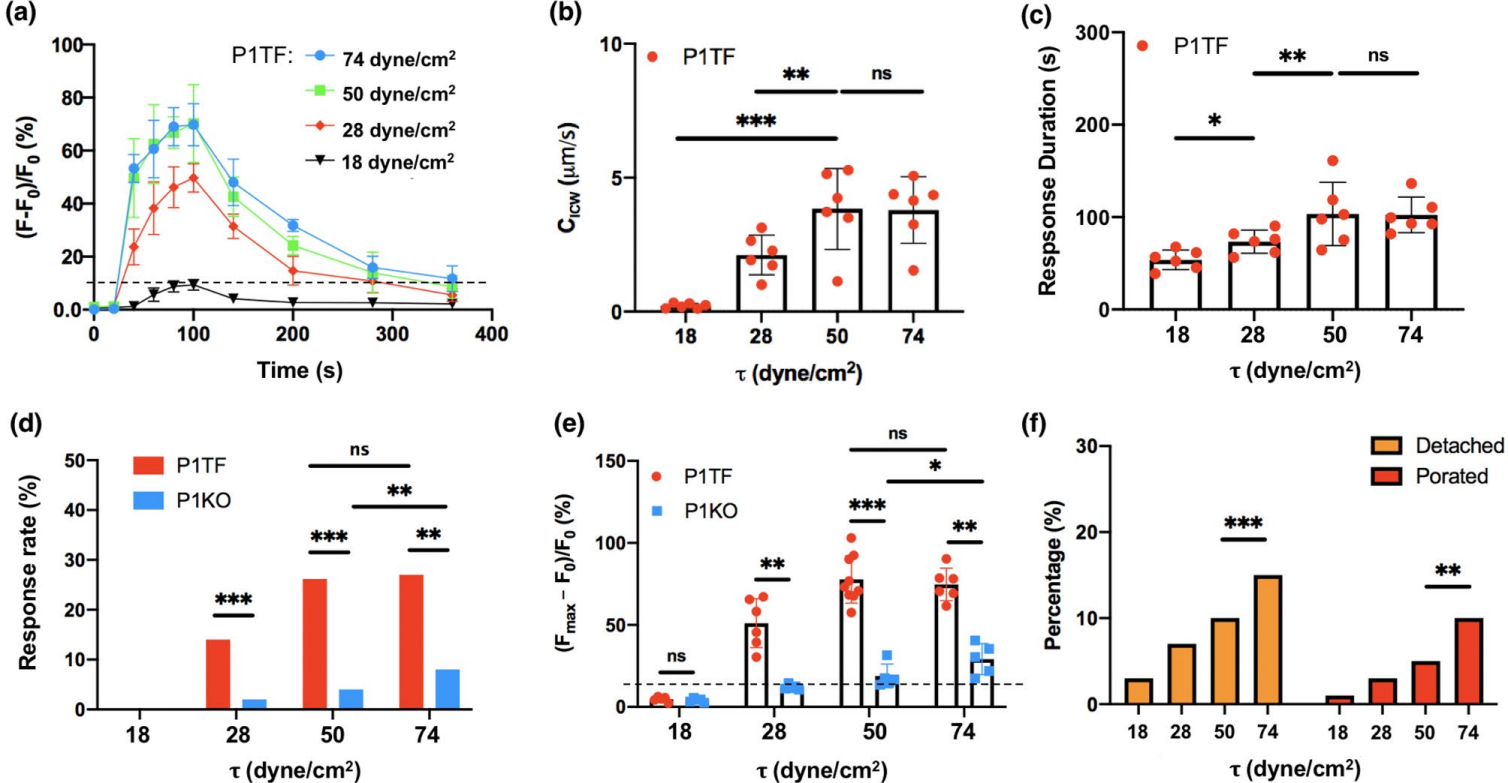
Piezo1 activation probability, ${[{Ca}^{2+}]}_{i}$ characteristics, and the percentages of cell detached and membrane porated show a shear stress-dependency under PL = 100 ms. (**a**) The average temporal intracellular calcium ratio profile of P1TF cells in response to insonification at 100 ms PL, 20% DC, 60 s TT but different shear stress levels. n = 6 for each data point, error bars represent $\pm \, \mathrm{ std}$. (**b**) Intracellular calcium wave speed (*C*_*ICW*_) in P1TF cells at different shear stress levels. n = 6, *p < 0.05, **p < 0.01, ***p < 0.001, and ‘ns’ denotes not significant. Two-tailed t test. (**c**) Response duration in P1TF cells at different shear stress levels (n = 6). (**d**) Responsive rate of P1TF and P1KO cells at various shear stress levels (n ~ 20 for each bar). (**e**) Peak normalized intracellular calcium ratio change in P1TF and P1KO cells at various shear stress levels (n ~ 6 for each case). Note: the data presented in (**a**) to (**e**) were based on cells that were neither detached nor porated following insonification. (**f**) Percentage of detached and porated cells over all the cells in the insonified region during the experiment at different shear stress levels (total number of cells analyzed at each shear stress level in the experiment: N ~ 80). Two-tailed t test in (**b**), (**c**) and (**e**), Fisher’s exact test in (**d**) and (**f**). *p < 0.05, **p < 0.01, ***p < 0.001, and ‘ns’ denotes not significant.

Figure 3 shows the typical ${[{Ca}^{2+}]}_{i}$ response elicited in individual P1TF cells without significant morphological change, detachment or PI uptake under four different levels of $\uptau$ = 18, 28, 50, and 74 dyne/cm^2^, respectively. US pulses were applied with 100 ms PL, 20% DC and 60 s TT, a protocol previously demonstrated to elicit a robust ${[{Ca}^{2+}]}_{i}$ response with about 70% ratio change^28^. In general, despite the random variations in the shape and orientation of the cells in the target area, the ${Ca}^{2+}$ response was mostly initiated at the leading edge of the cell (indicated by the white arrow) facing the acoustic streaming-induced shear stress (red arrow) and propagated toward the center (Supplementary Fig. S4). Quantitatively, stronger ${Ca}^{2+}$ response was elicited by higher shear stress (Fig. 4a), reaching a plateau after 50 dyne/cm^2^. This result is presumably correlated with the large deformation of the cell membrane and resultant opening of Piezo1 and other mechanosensitive (MS) ion channels in the highly stressed regions^24, 28^. Moreover, we calculated the intracellular ${Ca}^{2+}$ wave speed (*C*_*ICW*_) propagating from the initiation site (region ①) to the center of the cell (region ②) using small-region analysis^24^. The *C*_*ICW*_ initially increased with $\uptau$ before flattening out at ~ 3 μm/s beyond 50 dyne/cm^2^ (Fig. 4b). Also, the ${[{Ca}^{2+}]}_{i}$ response duration, measured by the full-width at half maximum (FWHM), initially increased with $\uptau$ and then plateaued at ~ 95 s beyond 50 dyne/cm^2^ (Fig. 4c). These results suggest that ${Ca}^{2+}$ influx through Piezo1 ion channels might reach saturation with increased membrane tension^27, 34, 35^.

Comparing between the P1TF and P1KO groups, no substantially detectable ${Ca}^{2+}$ response (with ${[{Ca}^{2+}]}_{i}$ change exceeding 10% of the baseline value was elicited at $\uptau$ = 18 dyne/cm^2^ in either cell type (Fig. 4d). At ≥ $\uptau$28 dyne/cm^2^, the P1TF cells were found to have a ${Ca}^{2+}$ response rate at least threefold higher (Fig. 4d) and a peak ${[{Ca}^{2+}]}_{i}$ twofold greater than the P1KO cells (Fig. 4e), indicating that Piezo1 is primarily responsible for transducing the shear stress. At even higher $\uptau$ from 50 to 74 dyne/cm^2^, only the P1KO cells showed further increase in the ${Ca}^{2+}$ response rate and peak ${[{Ca}^{2+}]}_{i}$ value, while the response in the P1TF cells saturated. However, under such high $\uptau$, both the rate of cell detachment and membrane poration in the non-detached cells increased significantly (Fig. 4f), leading to a higher risk of cell injury^36^. For example, as $\uptau$ increased from 50 to 74 dyne/cm^2^, the rate of cell detachment and membrane poration was elevated by 54% and 96%, respectively.

### Effects of PL on Piezo1 activation and ${[{{\rm{C}}{\rm{a}}}^{2+}]}_{{\rm{i}}}$

Next, we assessed the effects of PL on Piezo1 activation and ${[{Ca}^{2+}]}_{i}$ response at $\uptau$ = 50 dyne/cm^2^ by varying PRF in the range of 0.2 Hz to 20 kHz while keeping the other US exposure parameters constant (i.e., 20% DC and 60 s TT). This strategy allowed us to adjust PL in a broad range from 0.01 to 1000 ms, which covers the full spectrum of the PL used in US neuromodulation applications^12^. Under such insonification conditions, negligible temperature rise (< 1 °C, see Supplementary Fig. S5), cell detachment and membrane poration (< 10%) were produced (Fig. 4f) while effective Piezo1 activation could be elicited.

**Figure 5:**
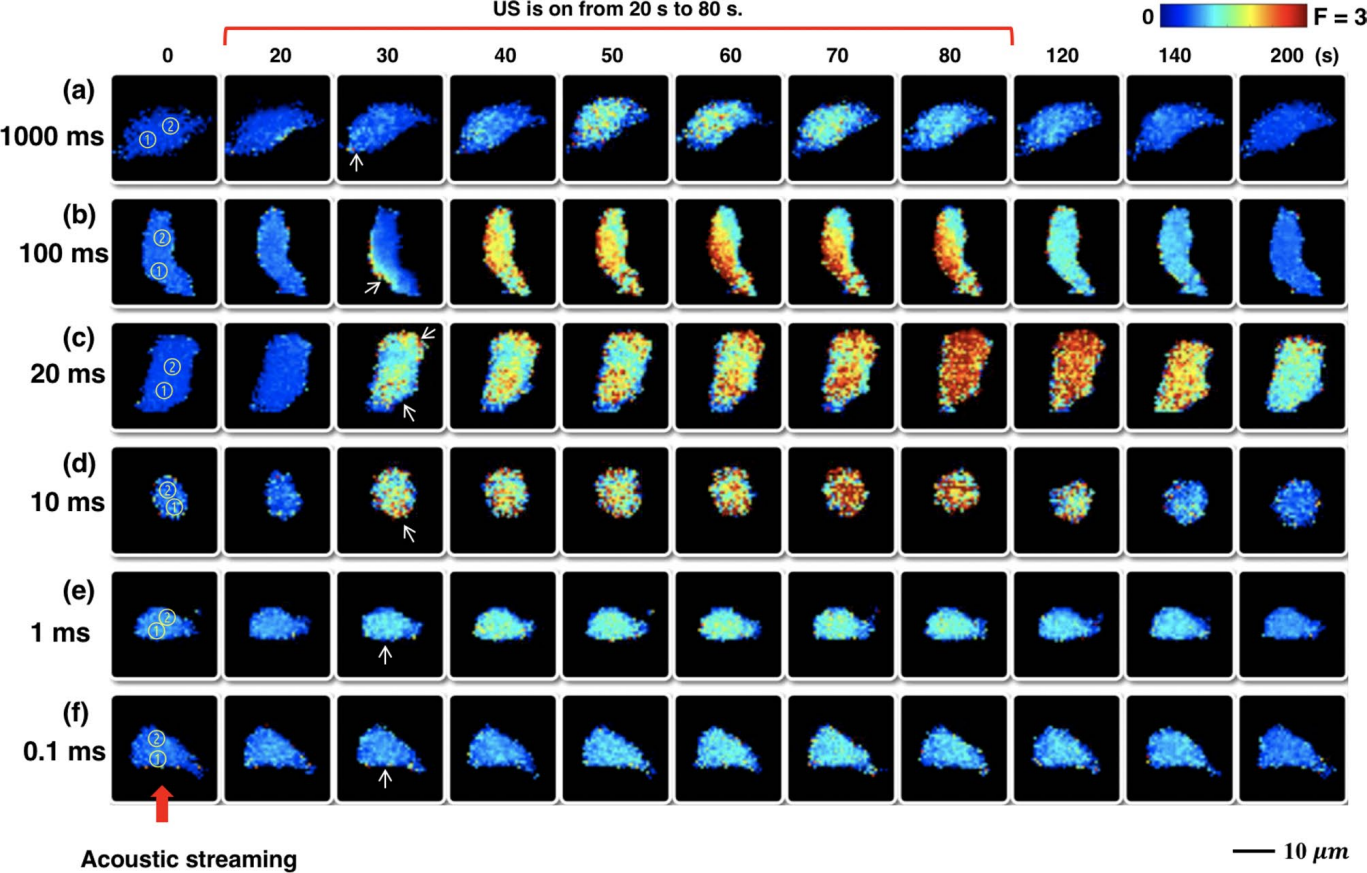
Sequential images show the temporal ${[{Ca}^{2+}]}_{i}$ ratio change in individual P1TF cells in response to various PL. (**a**) PL = 1000 ms. (**b**) PL = 100 ms. (**c**) PL = 20 ms. (**d**) PL = 10 ms. (**e**) PL = 1 ms. (**f**) PL = 0.1 ms. Circles with numbers represents the regions used for calculating *C*_*ICW*_. US was on from 20 to 80 s. White arrows indicate the ${[{Ca}^{2+}]}_{i}$ response initiation sites. Red arrow at the bottom indicades the direction of VD-SAW transducer generated acoustic streaming.

In general, the shear stress-elicited ${Ca}^{2+}$ response in the P1TF cells was found to be highly sensitive to PL with the strongest ${[{Ca}^{2+}]}_{i}$ change observed between 10 and 100 ms (Fig. 5). Outside this range at either shorter or longer PL, the elicited ${[{Ca}^{2+}]}_{i}$ response became much weaker despite that the same total acoustic energy was delivered to the cells. Similar to the observations at different $\uptau$ levels (Fig. 3), the initiation of ${[{Ca}^{2+}]}_{i}$ elevation started at the leading edge (Fig. 5a,b,d–f) or from both the leading and trailing edges of the cell (Fig. 5c) along the flow direction, and propagated toward the center. Additional experiments using the P1TF cells treated by either Thapsigargin or in calcium-free medium (Supplementary Fig. S6) indicated that this process is likely facilitated by a calcium-induced calcium release (CICR) mechanism whereby sufficient ${Ca}^{2+}$ influx from the highly stretched plasma membrane region triggers the ${Ca}^{2+}$ release from neighboring endoplasmic reticulums (ERs) in the cytosol, and such a diffusion–reaction process is repeated during insonification to propagate the intracellular calcium signaling^24, 37^.

**Figure 6:**
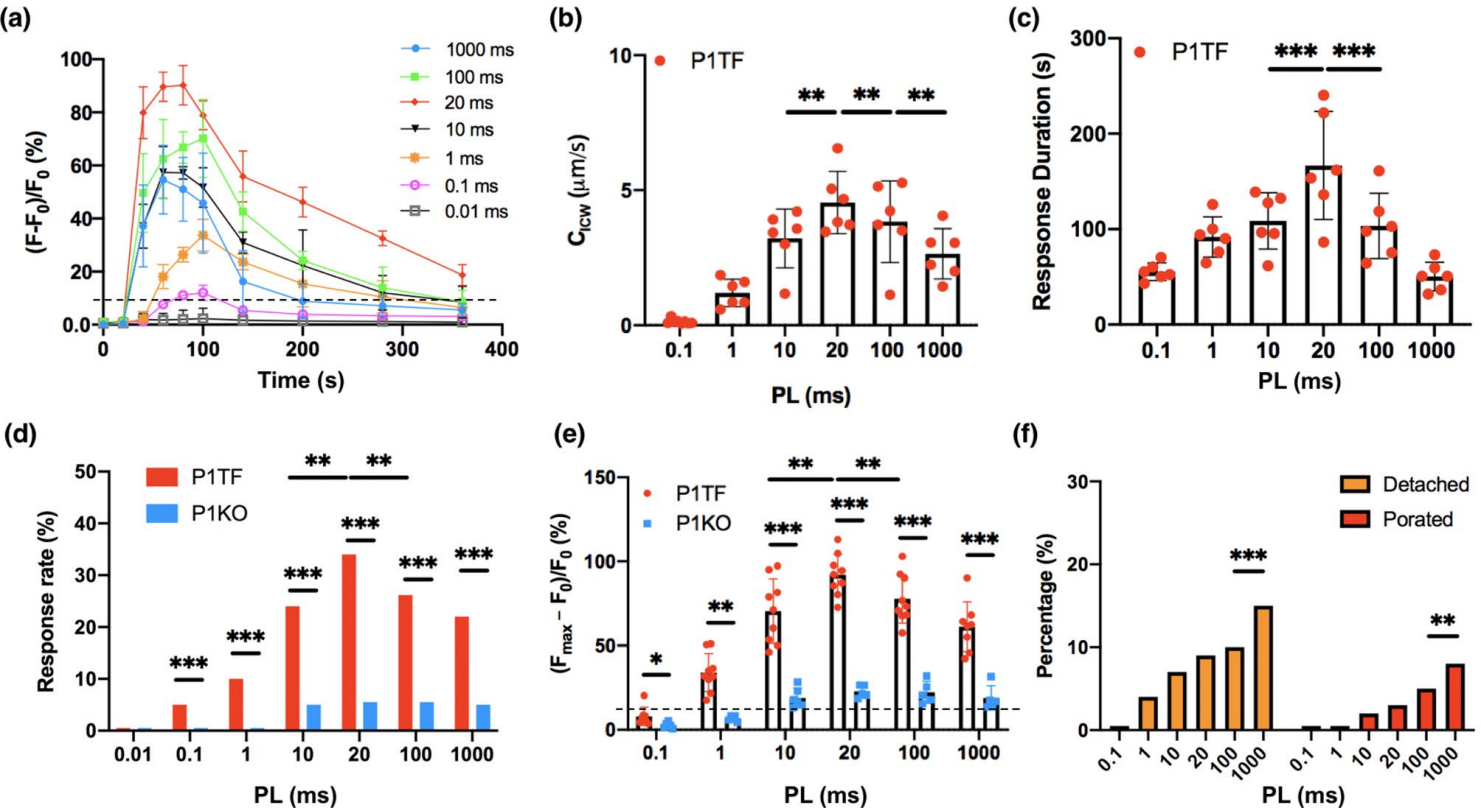
Piezo1 activation probability, ${[{Ca}^{2+}]}_{i}$ characteristics, and the percentages of cell detached and membrane porated show a PL dependence at a constant shear stress level ($\uptau$ = 50 dyne/cm^2^). (**a**) The average temporal intracellular calcium ratio profile of P1TF cells in response to insonification at $\uptau$ = 50 dyne/cm^2^, DC = 20%, TT = 60 s but different PL. n = 6 for each data point, error bars represent $\pm \mathrm{std}$. (**b**) Intracellular calcium wave speed (*C*_*ICW*_) in P1TF cells at different PL (n = 6). (**c**) Response duration in P1TF cells at various PL (n = 6). (**d**) Response rate of P1TF and P1KO cells at various PL (n ~ 20 for each bar). (**e**) Peak normalized intracellular calcium ratio change in P1TF and P1KO cells at various PL (n ~ 6 for each case). Note: the data presented in (**a**) to (**e**) were based on cells that were neither detached nor porated following insonification. (**f**) Percentage of detached and porated cells over all the cells in the experiment at different PL. (total number of cells analyzed under each condition: N ~ 80). Two-tailed t test in (**b**), (**c**) and (**e**), Fisher’s exact test in (**d**) and (**f**). *p < 0.05, **p < 0.01, ***p < 0.001, and ‘ns’ denotes not significant.

Quantitatively, Fig. 6a shows the average temporal profiles of ${[{Ca}^{2+}]}_{i}$ change in the PITF cells elicited at different PLs. The strongest ${[{Ca}^{2+}]}_{i}$ ratio change, up to nearly 90% above the baseline value, was produced by PL = 20 ms. Interestingly, the C_ICW_ and ${[{Ca}^{2+}]}_{i}$ response duration were also peaked at PL = 20 ms (Fig. 6b,c), and so were the response percentage and peak normalized ratio change in ${[{Ca}^{2+}]}_{i}$ (Fig. 6d,e). Altogether, these findings suggest that a unified mechanism may exist underlying this optimal insonification condition to produce the strongest ${[{Ca}^{2+}]}$ response. It is also worth noting that at PL = 20 ms, the response percentage (~ 30%) of the P1TF cells is sixfold of the value (~ 5%) in the P1KO cells (Fig. 6d). Similarly, the normalized ratio change of ${[{Ca}^{2+}]}_{i}$ (93.5%) in the P1TF cells is about fourfold of the value (24.6%) in the P1KO cells (Fig. 6e), indicating the much higher specificity and sensitivity of Piezo1 to shear compared to any other MS ion channels exist in the HEK293T cell membrane. In addition, the percentage of cell detachment and non-repairable membrane poration were found to increase monotonically with PL (Fig. 6f), presumably due to breakage of adhesive receptor–ligand bonds between the cell membrane and extracellular matrix^38^ and rupture of cell membrane under sustained shear with long stretch durations^39^. Therefore, prolonged increase in PL beyond 20 ms during insonification will result in higher risk of cell detachment and membrane poration without enhancing the ${Ca}^{2+}$ response in the target cells.

### Effects of US parameters on cell displacement (or deformation)

The activation of Piezo1 is known to be directly linked with the cell membrane deformation produced under tension^27^. Yet, the individual and accumulated membrane deformation produced by different US pulsating protocols have not been thoroughly investigated. To ascertain the effects of US parameters on cell membrane deformation, we analyzed the displacement of the maximum fluorescent intensity (380 nm) point over the entire cytosol during insonification as a surrogate marker related to cell deformation using a previously developed protocol^28^.

**Figure 7:**
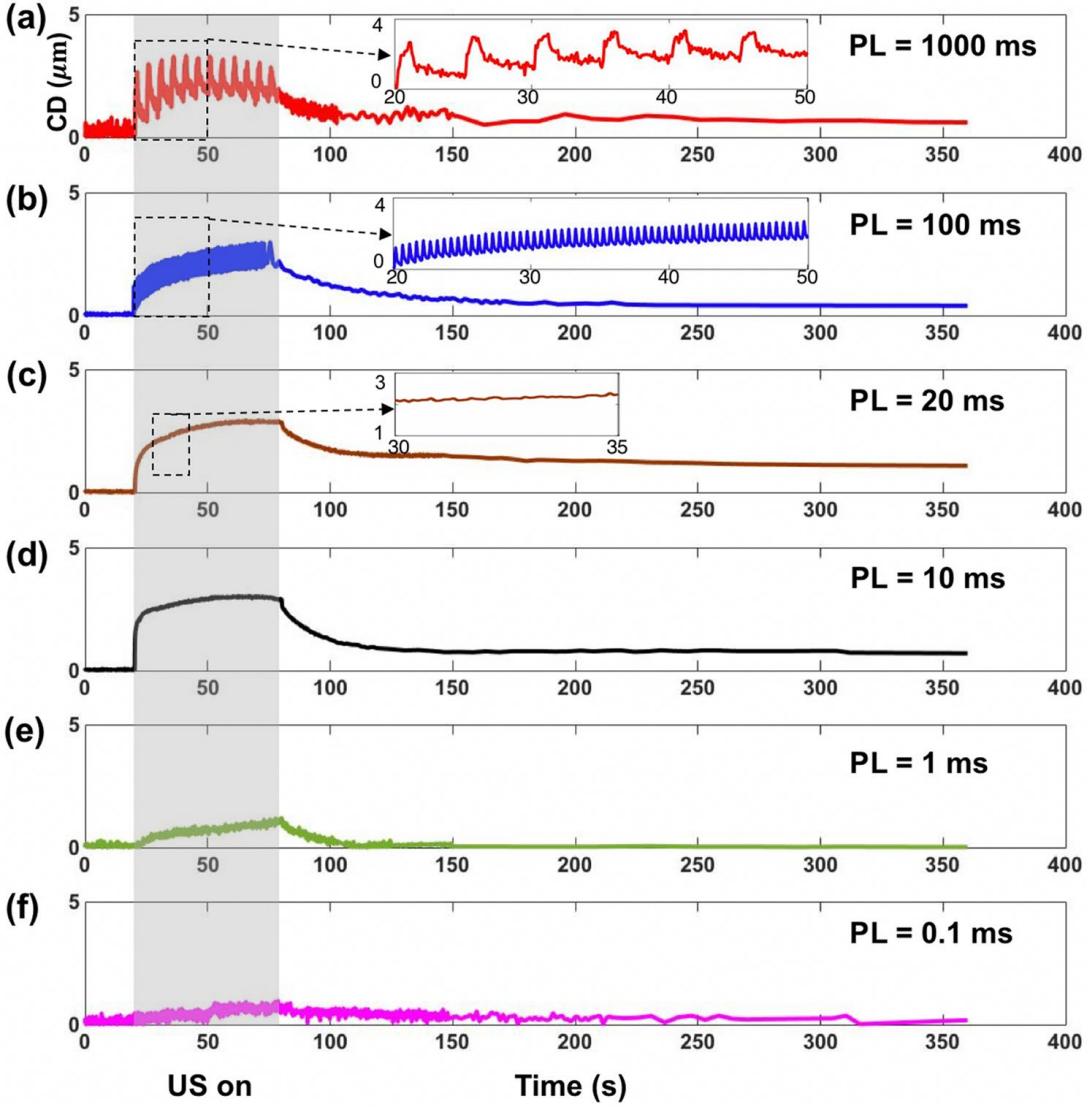
The time course of cell displacement (CD) shows distinct features at different PL, when $\uptau$ = 50 dyne/cm^2^, DC = 20%, TT = 60 s. (**a**) PL = 1000 ms. (**b**) PL = 100 ms. (**c**) PL = 20 ms. (**d**) PL = 10 ms. (**e**) PL = 1 ms. (**f**) PL = 0.1 ms. Gray rectangular indicate the time-window of insonification (20 s to 80 s).

Figures 7 shows representative results of cell displacement in response to different PLs produced at $\uptau$ = 50 dyne/cm^2^. Overall, the cell displacement (CD) vs. time curves showed a creep-recovery response characteristic of biological tissue and cells (i.e., viscoelastic soft materials) subjected to impulses of mechanical stress of constant amplitude^16, 40^. Much pronounced response was observed at PL ≥ 10 ms (Fig. 7a–d), which consisted of a rapid elastic deformation initiated at the onset of insonification (20 s), followed by a graduate viscoelastic response to reach the maximum cell displacement (CD_max_) during the insonification period. After the cessation of insonification (80 s), the cell displayed instantaneously a small elastic recovery, followed by a gradual yet significant viscoelastic recovery with an exponential decay time constant on the order of 40 s. Even after 200 s, substantial residual (creep) displacement of the cell could still be observed. In contrast, at PL ≤ 1 ms, weak and negligible response was observed (Fig. 7e,f). These results clearly demonstrate a PL dependent mechanical deformation of the cells during insonification. At the longest PL of 1000 ms, which corresponds to an PRP = PL/DC (or interpulse time) of 5 s, the creep-recovery response after each individual pulse could be resolved at the 10 fps frame rate used for fluorescent imaging (see inset of Fig. 7a). As PL and thus interpulse time decreased, the viscoelastic response and recovery times would diminish significantly, leading to a much more rapid accumulation of the overall response, largely associated with the elastic deformation and recovery produced by individual pulses (see inset of Fig. 7b). At PL = 10 ~ 20 ms, the accumulated CD during insonification reached the maximum with small oscillations between pulses associated with viscoelastic recovery. At further shortened PL ≤ 1 ms, however, much weaker response with slow accumulations were produced despite the higher number of pulses generated under such insonification conditions.

**Figure 8:**
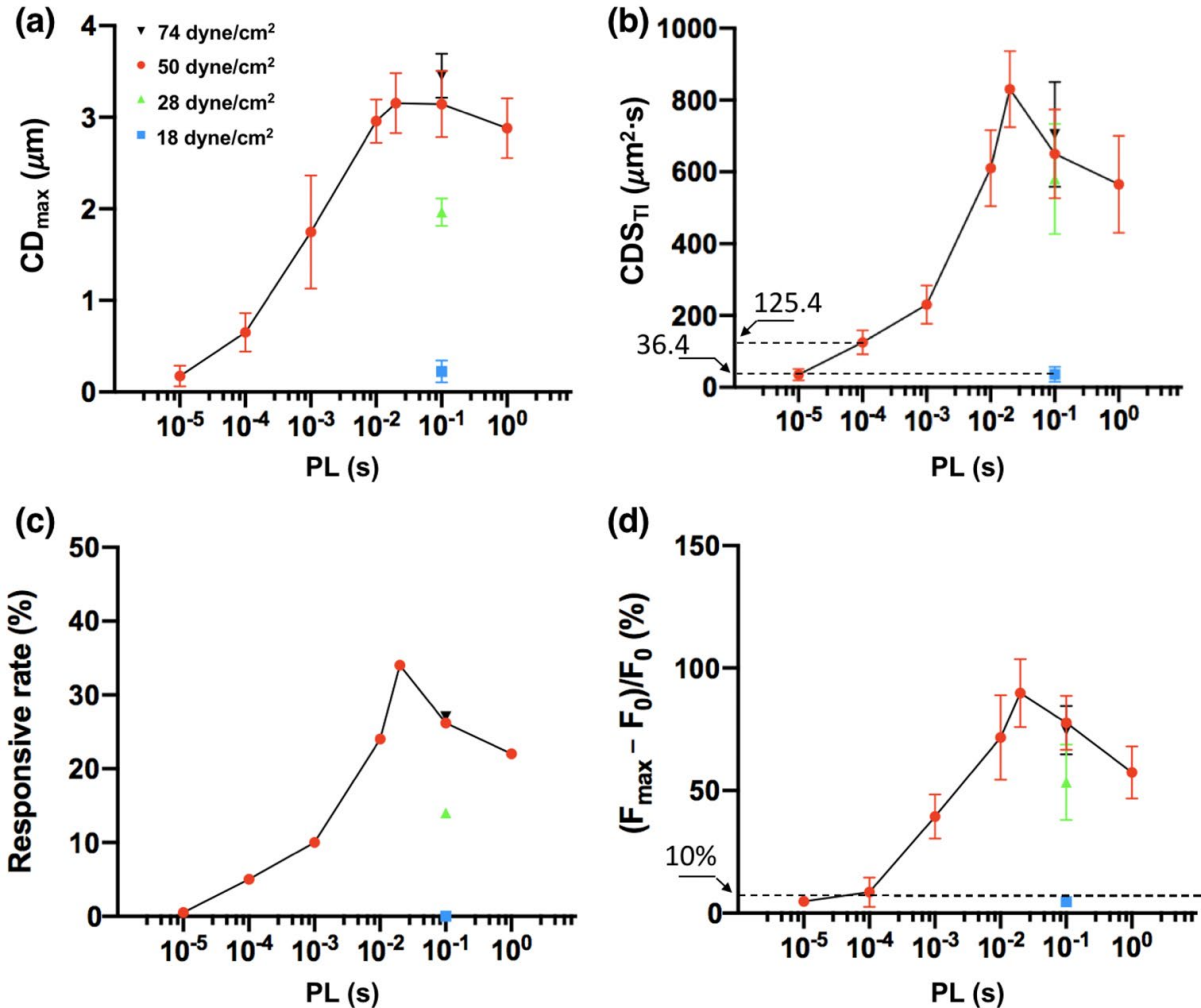
The time integral of cell displacement square (CDS_TI_) is a better predictor of the Piezo1 responsive rate and ${[{Ca}^{2+}]}_{i}$ uptake, compared to CD_max_. (**a**) Maximum cell displacement (CD_max_) during the insonification at different PL and $\uptau$. n = 6 for each condition. (**b**) Time integral of cell displacement square (CDS_TI_) during the insonification at different PL and $\uptau$. n = 6 for each condition. (**c**) Responsive rate of P1TF cells at different PL and $\uptau$. (**d**) Peak normalized intracellular calcium ratio at different PL from 0.01 ms to 1000 ms and under various $\uptau$ levels: 18 dyne/cm^2^ (blue square), 28 dyne/cm^2^ (green upward triangle), 50 dyne/cm^2^ (red circles) and 74 dyne/cm^2^ (black downward triangle). n = 6 for each condition.

Figure 8a illustrates the relationships between CD_max_ and PL produced under $\uptau$ = 50 dyne/cm^2^. As PL increases, the average CD_max_ will rise from 0.2 to 3.1 μm, reaching a peak at PL = 20 ms before decaying slightly thereafter. Since cell deformation and resultant ${Ca}^{2+}$ response to high strain-rate shear loading depend on both the amplitude and duration of the stimulation^24, 39^, we further calculated the time integral of cell displacement square (CDS_TI_) produced by insonification, which is presumably proportional to the strain energy density impulse produced on the cell membrane^39^. As shown in Fig. 8b, CDS_TI_ appears to reveal a biphasic dependence on PL with a distinct peak achieved at 20 ms. At shorter PL, CDS_TI_ increases progressively with PL. After reaching the maximum at PL = 20 ms and at longer PL, CDS_TI_ will decrease at a different rate. In comparison to CD_max_, the profile of CDS_TI_ appears to better resemble the profiles in the responsive rate and peak ${[{Ca}^{2+}]}_{i}$ ratio change of the P1TF cells at different PLs (Fig. 8c,d). Furthermore, among all the tests, the highest response rate with associated strongest ${[{Ca}^{2+}]}_{i}$ increase was produced at PL = 20 ms under $\uptau$ = 50 dyne/cm^2^, which is consistent with the peak in CDS_TI._ In contrast, although the maximum CD_max_ were produced at PL = 100 ms under $\uptau$ = 74 dyne/cm^2^, lower than peak response rate and ${[{Ca}^{2+}]}_{i}$ increase were observed, indicating that CD_max_ alone is not reliable for predicting the ${[{Ca}^{2+}]}_{i}$ response. Finally, it is important to note that CDS_TI_ (125.4 s ${\mu m}^{2}$) at PL = 0.1 ms is significantly higher than the value (35.4 s ${\mu m}^{2}$) at PL = 0.01 ms under $\uptau$ = 50 dyne/cm^2^, and also higher than the corresponding value (36.4 s ${\mu m}^{2}$) at PL = 100 ms under 18 dyne/cm^2^. These results suggest that a threshold in CDS_TI_ (36.4 s *μm*^2^· < CDS_TI_ < 125.4 s *μm*^2^) is likely required to activate Piezo1. Overall, our results confirm the important role of cell deformation in Piezo1 activation, and more importantly, suggesting that fine-tuning PL to match with the intrinsic viscoelastic properties of the cell may significantly improve the efficiency in ultrasonic activation of Piezo1.

### Numerical modeling of four-state channel gating dynamics of Piezo1 in relation to PL-dependent ${[{Ca}^{2+}]}_{i}$ response

**Figure 9:**
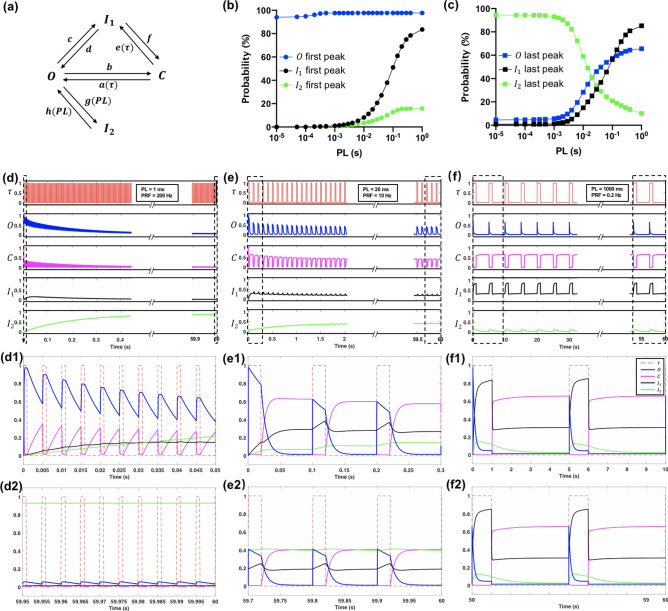
Simulation of Piezo1 channel kinetics by the modified four-state gating model. (**a**) A schematic of the modified four-state gating mechanism for Piezo1, with arrows indicating transitions between open (O), closed (C) and two inactivated states (I_1_ and I_2_). In the original model, $a\left(\uptau \right)\mathrm{ and }e(\uptau )$ are coupling coefficients between C, O and I_1_ states, respectively, which are modulated by the pressure (or equivalently $\uptau$ in this study) applied to the plasma membrane, leading to membrane tension. Moreover, *g* and *h* are the coupling coefficients between O and I_2_ states, which are constants. In the modified model, *g* (*PL*) and *h* (*PL*) are assumed to be PL-dependent while the rest of the coefficients are the same as in the original model. (**b**) The peak probability of the O, I_1_ and I_2_ evoked by the 1^st^ pulse at various PL. (**c**) The peak probability of the O, I_1_ and I_2_ evoked by the last pulse at various PL. (**d**–**f**) The normalized shear stress (red), simulated Piezo1 open (blue), closed (magenta), inactivation 1 (black) and inactivation 2 (green) probabilities change in response to 60 s insonification for PL = 1, 20 and 1000 ms, respectively. (**d1**–**f1**) The magnified plot at the beginning of the insonification for PL = 1, 20 and 1000 ms, respectively. (**d2**–**f2**) The magnified plot toward the end of the insonification for PL = 1, 20 and 1000 ms, respectively.

To facilitate data interpretation from the experiments, we have adapted a four-state model for channel gating of Piezo1 constructed based on electrophysiology measurements^27^. Especially, the model can be used to reveal the intricate dynamic interactions between the open (O), closed (C) and two inactivation (I_1_ and I_2_) states of the Piezo1 channel under dynamic loading that determines its overall frequency response (Fig. 9a). Most importantly, previous studies^27^ have uncovered two kinetically distinct inactivation (i.e., non-conducting despite continued presence of membrane tension) states of Piezo1 with drastically different current recovery time constants (24 ms for I_1_ and 10 s for I_2_). As a result, Piezo1-mediated current influx may rise rapidly (~ ms) in response to membrane tension and then decay exponentially over a much longer timescale due to inactivation^17^. Because of inactivation, Piezo1 will respond strongly to the first (or onset of the) stimulus when subjected to repetitive membrane tension, and gradually become desensitized in a frequency filtering process highly modulated by the waveform and PRF of the stimulus^27^. Since Piezo1 exhibits cationic nonselective permeability^17^, we hypothesize that the inactivation of Piezo1 may affect the ${Ca}^{2+}$ influx induced by US pulses in a PL-dependent manner.

In the original four-state gating model, the transition rates $a$($\uptau )$ and $\mathrm{e}$($\uptau )$ were assumed to be modulated by the amplitude of the micropipette pressure applied to the plasma membrane (or equivalently shear stress in this study). Other transition rates in the model were constants, which were determined by numerical fitting of the experimental data in response to 2–20 Hz stimuli. However, we found that the original four-state model is insufficient for capturing the trend in the time-integral of the evoked current (Supplementary Fig. S8c). To overcome this limitation, we further assume that the transition rates between O and I_2_ (*g* and *h*) are PL dependent (see Eqs. S1 and S2 in SI) while keeping all the other parameters unchanged. This modified four-state model has been shown to capture well both the transient and integrated features of Piezo1 kinetics (Supplementary Fig. S8b,c).

The general features of Piezo1 have been recapitulated under the insonification conditions evaluated in this study. Figure 9 shows the time course of the response probability of the four states (O, C, I_1_ and I_2_) of the Piezo1 channels on the cell membrane during insonification for PL = 1, 20 and 1000 ms. Assuming constant $\uptau$ (or membrane tension) during each pulse, the model simulation suggests that the Piezo1 channel open probability will increase rapidly to the maximum during the “on time” of the first pulse, and then decay exponentially in the “off time” of the US. Such a process is repeated following each of the subsequent pulses, leading to a progressively decayed response during the entire insonification process. The change in the Piezo1 channel “open” probability is accompanied by an inversely varied “closed” probability, and further modulated concomitantly by the two “inactivation” states. In particular, the time profiles of the four states were found to be highly sensitive to PL. At short PL (= 1 ms), the probability of O increases quickly to the maximum peak (98%) near the end of the first pulse while the probability of I_1_ only elevates incrementally, reaching the maximum (16%) after about ten pulses before decaying slowly to the ground level toward the end of insonification. More interestingly, the probability of I_2_ increases progressively and accumulates significantly during the 12,000 pulses, reaching the maximum (90%) near the end of the insonification to desensitize most of the Piezo1 channels. Therefore, the significant and predominant rise in I_2_ limits the ${Ca}^{2+}$ response elicited by short PL. In contrast, at long PL (= 1000 ms), the probability of O increases instantaneously at the beginning of the first pulse to the maximum peak (98%) and then decays quickly to near ground level (< 3%) before the cessation of the pulse. During this period, both I_1_ and I_2_ are evoked fully to reach their corresponding peaks of 82% and 16%, respectively. In the ensuing long inter-pulse time (4000 ms), the levels of I_1_ and I_2_ will decrease yet still maintaining between 5 to 25%. As a result, the probability of O evoked by the subsequent pulses will drop below 65% and reminds fairly constant during the insonification process. The inefficient opening during each pulse, combined with the limited total number of pulses (12) during insonification lead to low ${Ca}^{2+}$ response produced by long PL. At the optimal PL = 20 ms, the probability of O expands the full pulse length while the levels of I_1_ and I_2_ are kept below 50% during the insonification process. The combination of efficient opening during each pulse with a modest decay of the peak opening rate and sufficiently large number of pulses (600) during the insonification produces a strong ${Ca}^{2+}$ response.

**Figure 10:**
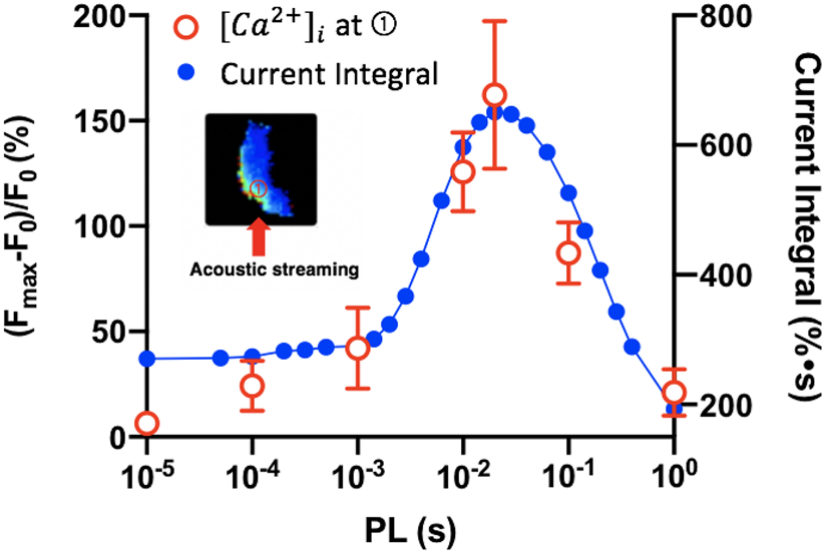
Correlation of the simulated current integral with the experimentally measured ${Ca}^{2+}$ influx at the initiation site. Red open circles: peak normalized ${[{Ca}^{2+}]}_{i}$ peak change near the cell boundary at region ① in experiment (n ~ 6 at each PL). Blue closed circles: simulated time-integral of current obtained by the modified four-state model.

To compare with the ${[{Ca}^{2+}]}_{i}$ change observed in the experiment, we plot the current integral on the cell membrane under tension during insonification calculated by the modified four-state model for different PLs. To match with the model simulation with minimal influence of CICR in the cytosol, we analyzed the ${\left[{Ca}^{2+}\right]}_{i}$ ratio change near the cell boundary in region ① of each cell where the calcium response was initiated (Figs. 3, 5). As shown in Fig. 10, the simulation results demonstrate a similar biphasic dependency on PL with a peak achieved at PL = 20 ms, as observed from the experiments. This reasonably good agreement between modeling prediction and experimental measurement further supports the notion that inactivation states of Piezo1 play an important role in shaping its optimal response condition to US simulation.

## Discussion

Sonogenetics represents a promising approach for non-invasive and selective activation of target cells in the body that are genetically modified to overexpress a specific MS ion channel^23^. A critical step in successful sonogenetics applications is to determine the appropriate insonification conditions that are most effective for activating the target MS ion channel while minimizing adverse effects. In this study, we utilized a novel VD-SAW platform to investigate the optimal insonification parameters for Piezo1 activation and ${[{Ca}^{2+}]}_{i}$ response in HEK293T cells transfected with Piezo1. Under the same insonification DC, total acoustic energy and treatment time (60 s), we have discovered that Piezo1 activation depends critically on PL, with a distinct peak produced at PL = 20 ms under a constant shear stress of 50 dyne/cm^2^ (i.e., the highest Piezo1 activation probability with associated strongest ${[{Ca}^{2+}]}_{i}$ response at < 10% cell detachment or membrane injury).

In general, multiple factors at three different levels may contribute to the PL-dependency in Piezo1 activation by US. First, the transmission and penetration depth of US in the body are frequency dependent and the resultant tissue deformation (produced either by acoustic radiation force or shear) will be influenced by the acoustic and viscoelastic properties of the interposing tissues between the US transducer and the target organ^41–43^. Even at the superficial skin level, the mechanical filtering effects of the tissue to insonification have been demonstrated, for example, in the touch receptor neurons in *C. elegans*^40^. Second, in the target tissue, the response to insonification at the cellular level will be influenced by the viscoelastic properties of the cytoskeleton and plasma membrane. Actin filaments, microtubules, and intermediate filaments in the cytoskeleton form an adaptive network that can resist stress-induced deformation in order to protect the intracellular compartments^44^. Similarly, the lipid bilayer of the plasma membrane is also known to have its own distinct viscoelasticity properties^45^, which influence lipid-protein or protein–protein interaction-induced cell signaling across the membrane surface^46^. Altogether, the convolution of the viscoelastic properties of the tissues and target cells will impose a time-dependent filtering effect on the mechanical forces delivered by the US pulses. As a result, only a narrow range of the PL (determined by the combination of DC and PRF) around the peak region of the viscoelastic response spectrum of the tissue-cell complex may be used to produce effective deformation and thus eliciting a robust calcium response in the target cell during insonification without causing significant adverse effects (e.g., over-heating of the interposed tissues or injuring the target cells). In this study, such a PL-dependency in cell deformation is exemplified by the time-integral of cell displacement square (CDS_TI_), which shows a biphasic relationship with PL, and correlating with the Piezo1 activation probability and ${[{Ca}^{2+}]}_{i}$ response (see Fig. 8).

Beyond the filtering effects of tissues and cells, a third level of factors that can critically influence the calcium (and thus neural) response of the target cell to insonification is the kinetics of the MS ion channels on the plasma membrane under mechanical deformation. Such a factor was not examined in previous sonogenetics studies^18, 23^. In comparison, recent patch-clamp electrophysiology studies have demonstrated the importance of inactivation of Piezo1 and other MS ion channels in modulating cellular physiology^17, 27^. In the previous electrophysiology studies, however, PL was fixed at 30 ms while the PRF was varied from 0.5 to 50 Hz, leading to different DC and total mechanical energy applied to the cell membrane^27^. In this work, we kept the DC, TT and thus the total acoustical energy delivered to the target cells constant to order to determine the optimal PL for safe and effective activation of Piezo1. The experimental observations were supported by the simulation results from the modified four-state model for Piezo1 channel gating, further reinforcing the idea that optimal US protocols could be developed in sonogenetics based on the characteristics of the target MS channels determined by their intrinsic structures^47^.

Moreover, our results suggest that the viscoelastic properties of the target cell are also important factors that will influence the mechanical response (see Fig. 7), and consequently, the resultant membrane tension that leads to Piezo1 activation. The coincident overlap in the peak of CDS_TI_ (Fig. 8b) and the model-predicted current integral of Piezo1 (Fig. 10) further accentuate the distinct maximum ${[{Ca}^{2+}]}_{i}$ response observed under PL = 20 ms. Conceptually, it is possible that the peaks for cell deformation and MS ion channel activation are generally achieved at different PLs, which would broaden the range of insonification conditions for neuromodulation yet with potentially reduced specificity and sensitivity.

In summary, we have demonstrated that PL is a critical parameter in ultrasonic activation of Piezo1 and resultant intracellular calcium response. By producing impulsive yet sufficiently sustained cell deformation using acoustic streaming-generated shear stresses, the Piezo1 ion channels embedded in the cell membrane could be opened. The effectiveness of Piezo1 activation is not achieved by rapidity of the stimuli or high number of repetitions using short PL (see also Supplementary Fig. S9) or high PRF for a fixed DC. Neither would it be achieved using long PL or low PRF without prolonging treatment time or increasing the risk of injury. For the same acoustic energy delivered to the target cells, therefore, the shear stress amplitude and PL of the insonification need to be optimized to achieve safe and efficient activation of Piezo1 based on the accumulated effects of adequate yet not excessive membrane deformation. The knowledge gained in this study has significant implications to sonogenetics applications, in which the PL of US should be carefully selected based on the properties of target tissue, cell and MS ion channels. Altogether, our work underscores the importance of fine-tuning the stimulus parameters to maximize the safety and efficacy of US neuromodulation strategies for noninvasive treatment of various neurological disorders.

## Methods

### Cell culture and transfection

HEK293T-P1KO cells (Piezo1 knock-out human embryonic kidney cells) and plasmid Mouse Piezo1-pIRES-EGFP in pcDNA3.1 were obtained from Dr. Jorg Grandl of Duke Neurobiology and previously described^48^. Cells were cultured in a 6-well plate in DMEM (high glucose) supplemented with 10% heat-inactivated fetal bovine serum (FBS) and penicillin/streptomycin antibiotics (DMEM complete medium), and grown in a cell culture incubator at 37 °C with 5% CO_2_ as previously described^17^. Cells were transiently transfected with Mouse Piezo1 (3 μg) in the presence of 10 μM ruthenium red using Fugene6 (Promega, Madison, WI) according to manufacturer protocol. About 20–30% of cells showed positive GFP expression indicating successful transfection of Piezo1 and were considered as P1TF cells. After 2 days, cells were reseeded in a 35 mm glass-bottomed Petri dish (81158, ibidi), which was coated with 50 μg/mL Fibronectin (ThermoFisher Scientific). Cells were then incubated in DMEM complete medium at 37 °C for 3 h before US treatment.

### VD-SAW transducer

A focused, 20-finger pair interdigital transducer (IDT) SAW device with aperture size (12 × 8 mm) was used to generate ultrasound pulses in the form of leaky pressure waves. The width of each finger and the gap between two adjacent fingers were set as $\lambda /4=32.5\, \mu m$ to define the SAW wavelength at $\lambda =130 \,\mu m$. The fingers were designed as a series of concentric-circular arcs with a 40$^\circ$ open angle to produce a focused acoustic filed. A 0.5-mm thick, 128$^\circ$-Y cut LiNiO3 wafer (Precision Micro-Optics) was used as the substrate. A double-layer metal (90 nm Au and 10 nm Cr) was subsequently deposited on the wafer using an e-beam evaporator (CHA solution), followed by a lift-off process to remove the photoresist and form the IDT. The fundamental frequency of IDT (33 MHz) was determined by a network analyzer prior to operation. Sinusoidal signals with pulse modulation were produced by a signal generator (Model 4065, B&K Precision), amplified by a 55 dB RF amplifier (Model A150, ENI), then applied to the IDT.

### Fluorescence and bright field imaging

Fluorescence and bright field imaging systems were incorporated in the VD-SAW platform to monitor the intracellular ${Ca}^{2+}$ signaling and characterize the morphological change of the cells produced by insonification. Intracellular calcium response (${\left[{Ca}^{2+}\right]}_{i}$) was measured by fluorescence imaging using the indicator dye fura-2 AM^49^. After incubation, the Petri dish was washed 3 times with OptiMEM to eliminate the excess fura-2 AM before insonification. 100 μg/mL propidium iodide (PI) was added to monitor membrane permeability change and cell necrosis^24^. A monochromator (DELTARAM X; PTI) was used for calcium and PI imaging. ${\left[{Ca}^{2+}\right]}_{i}$ was measured by ratiometric imaging with fura-2 at 340 and 380 nm excitation, and the fluorescence emission was recorded at 510 nm by a sCMOS camera (EDGE 5.5 CL; PCO) at a frame rate of 10 Hz for a total recording time up to 350 s as described previously^28^. Thereafter, the ratio between fluorescence intensity from 340 and 380 nm excitation (F) over time was obtained in the EasyRatioPro 2 imaging processing software (HORIBA Scientific).

### Data analysis

The normalized fluorescence ratio change [($$F-{F}_{0})/{F}_{0}$$], where *F*_*0*_ is the average resting value of the cell before US treatment, was further analyzed using MATLAB (MathWorks) to obtain the characteristics of $${\left[{Ca}^{2+}\right]}_{i}$$ (peak amplitude and response time)^28^. The response time is defined as the full-width at half maximum of the $${\left[{Ca}^{2+}\right]}_{i}$$ response. At high input voltage (or shear stress), cells might move along the acoustic streaming direction during insonification, leading to some fluorescence intensity fluctuations, up to 10% of the resting value. Thus, only the cells with the peak normalized ratio change greater than 10% were considered as “responsive” above the background noise level^28^. The intracellular calcium wave (ICW) speed was calculated based on the protocol previously described^24^. Two circular regions (5 $$\mathrm{\mu m}$$ in diameter), with region 1 located at the area where $${\left[{Ca}^{2+}\right]}_{i}$$ initiated and region 2 near the center of the cell, were drawn manually by the ROI manager in ImageJ. The time delay of the half maximum of $${\left[{Ca}^{2+}\right]}_{i}$$ response at these two regions were used as the $${\left[{Ca}^{2+}\right]}_{i}$$ propagating time. The peak $${\left[{Ca}^{2+}\right]}_{i}$$ change at region 1 was further used as a measure of the $${Ca}^{2+}$$ influx mediated by Piezo1 in P1TF cells.

## Supplementary Information

Supplementary Information
