# Supplementary material for: Optimal pulse length of insonification for Piezo1 activation and intracellular calcium response
Source: Sci Rep. 2021 Jan 12;11:709. doi: 10.1038/s41598-020-78553-2 (PMC7804118; doi:10.1038/s41598-020-78553-2)
Supplement: Supplementary file 1 — Supplementary Information [file 41598_2020_78553_MOESM1_ESM.pdf]

Supplementary Information

Optimal Pulse Length of Insonification for Piezo1 Activation and Intracellular Calcium Response

Defei Liao[1], Ming-Yen Hsiao[2,3], Gaoming Xiang[1] and Pei Zhong[1]

[1] Department of Mechanical Engineering and Materials Science, Duke University, Durham, NC 27708 USA

[2] Department of Physical Medicine & Rehabilitation, National Taiwan University College of Medicine, Taipei, Taiwan

[3] Department of Physical Medicine & Rehabilitation, National Taiwan University Hospital, Taipei, Taiwan

*Correspondence to:

Pei Zhong
Department of Mechanical Engineering and Materials Science
Duke University
Box 90300
Durham, NC 27708, USA
(919) 660-5336 (voice)
(919) 660-8963 (fax)
pzhong@duke.edu (email)

SUPPLEMENTARY METHODS

Calibration of SAW vibration amplitude. To calibrate our VD-SAW transducer, we measured the vertical vibration amplitude (A) at the focus of SAW (f_0 = 33 MHz) produced by the IDT in air at different input voltages (V_{in}) using a custom-built heterodyne interferometer. As shown in Supplementary Fig. S1, a linear relationship between the vibration amplitude and IDT input voltage was observed, which is consistent with previous reports[1].

Measurement of pressure amplitude. The pressure amplitude in a water tank (Supplementary Fig. S2) generated by the VD-SAW transducer (V_{in} = 80 mV) at z = 3 mm plane was measured by a fiber optic probe hydrophone (FOPH-500, RP Acoustics). The hydrophone was controlled by a 3-axis positioning system with a step size of 0.5 mm and a scan range of 8 mm along x or y axis. The FOPH output was first recorded by a digital oscilloscope (LeCroy 9310A, Chestnut Ridge) and then analyzed in MATLAB.

Quantification of shear stress by numerical stimulation. We estimated the shear stress produced by the VD-SAW at different input voltages in the area of the target cells in the petri dish (see Supplementary Fig. S3). We constructed a numerical model in COMSOL. The 1st order acoustic fields (p_1 and v_1) are solved using the Thermoviscous Acoustics interface in the Acoustics Module. The vibration amplitude A on the IDT was used to determine the veloctiy boundary condition ($v = \omega \cdot A$, $\omega = 2\pi f_0$). The streaming flow (p_2 and v_2) is solved using the Laminar Flow physics interface of the CFD Module by adding the appropriate time-averaged 1st order sources: a mass source and a volume force. The shear stress produced by the streaming velocity in the target cell area is calculated by the

gradient of v_2 along the z axis: $\tau = \mu \frac{dv_2}{dz}$ (μ is the dynamic viscosity of water. At 20℃, $\mu = 1 \times 10^{-3} N \cdot s \cdot m^{-2}$).

Temperature change induced by insonification. A 0.2-mm bare-wire thermocouple (Physitemp Inc., Clifton, NJ) was used to measure the temperature change in the culture medium inside the Petri dish induced by VD-SAW at different PLs. The input voltage was 100 mV for all the measurements, and the maximum temperature elevation produced during the 60 seconds insonification was less than 1 ℃ (see Supplementray Fig. S5).

Examination of the source of intracellular calcium signaling. We performed additional experiments to examine the source of intracellular calcium response. We used 3 μM thapsigargin (Invitrogen™) in one group to discharge intracellular calcium stores before insonification at different PLs. We also used Ca^{2+}-free medium (chelated with EGTA) in another group during insonification. The experimental results are presented in Fig. S6.

Examination of the time-dependent acoustic parameters. To examine whether the buildup and decay of acoustic parameters are time-dependent, we simulated the transient SAW field in COMSOL using time-dependent (transient) solver. The computation was performed on a Duke virtual desktop with Intel Xeon CPU E5-2699 v4 @2.2 GHz 2 processors, 64-bit Windows 10, and 60 GB RAM. The time-dependent simulation took approximately 60 hours for two 10 μs pulses with 0.5 μs time step. We found that both p_1 and v_2 take about 1 μs to reach their steady states at the onset while fully decaying within 1 μs following the cessation of insonification. We concluded that the pulsating protocol in our setup has minimal effect on the buildup of acoustic energy. It is intuitive that acoustic energy is much more difficult to accumulate in a large domain (Petri dish: 35 mm in diameter) than for example in a confined micro-cavity (<1 mm characteristic length) as

demonstrated by other studies[2]. Therefore, the acoustic streaming-induced shear stress applied to the adherent cells could be considered as an ideal square wave (Supplementary Fig. S7).

Modified four-state Piezo1 gating model. We developed a modified four-state Piez1 gating model to explain the experimental observations in this study. The transition rates in the model are: $a(\tau) = 5.1 \times \exp(-\tau/6.8)$, $b = 116.9\ s^{-1}$, $c = 8.0\,s^{-1}$, $d = 4.0\ s^{-1}$, $e(\tau) = 34.6 \times \exp(\tau/6.8)\ s^{-1}$, and $f = 33.6\ s^{-1}$. These rates are the same as the original gating model[3]. We further assume that g and h will depend on the repetition frequency of the stimulus. For continues sinusoidal wave, repetition rate is the fundamental frequency (f_0). For pulsed wave, repetition rate is the pulse repetition frequency (PRF). By simultaneously fitting experimental data at frequency from 0.5 Hz to 50 Hz, we found g and h can be best fit by the following equations:

$$g(Freq.) = \alpha \cdot \left[1 - \beta \cdot \exp\left(-\frac{Freq.}{\gamma}\right)\right] \qquad (S1)$$

$$h(Freq.) = \frac{\delta}{g(Freq.)} \qquad (S2)$$

where $\alpha = 7.0\ s^{-1}, \beta = 0.7, \gamma = 44.8\ Hz$ and $\delta = 2.4$.

Compared to the experimental measurement of the membrane current mediated by Piezo1 in the same cell line[3], we found the modified four-state gating model is sufficient to capture the basic features of the first peak, last peak and tonic current (Supplementary Fig. S8b). More importantly, our modified model can recapitulate the frequency-filtering effect of Piezo1 on the current integral, whereas the original model does not (see Supplementary Fig. S8c). For pulsed waves with a given DC, g and h can be written as a function of PL:

$$g(PL) = \alpha \cdot \left[1 - \beta \cdot \exp\left(-\frac{DC}{PL \cdot \gamma}\right)\right] \quad (S3)$$

$$h(PL) = \frac{\delta}{g(PL)} \quad (S4)$$

SUPPLEMENTARY FIGURE CAPTIONS

Supplementary Fig. S1. The vibration amplitude produced by the 33 MHz IDT in air at different input voltages measured by heterodyne interferometry. Values are expressed as means ± std. (n = 3).

Supplementary Fig. S2. Characterization of the pressure field produced by the VD-SAW. (a) A representative pressure waveform of 2 μs pulse generated by the VD-SAW at V_{in} = 80 mV in a water tank measured at the focus (x = 6 mm, z = 3 mm) by a fiber optic probe hydrophone. (b) The corresponding normalized spectrum shows a peak frequency at 33 MHz. (c) Comparison of pressure amplitude along the x-axis between numerical simulation results in free field (top) and experimental measurements. (d) Pressure amplitude distribution in the free field measured by the fiber optical probe hydrophone in the z = 3 mm plane.

Supplementary Fig. S3. The shear stress produced by IDT in the target cell region in the petri dish at different input voltages estimated by COMSOL modeling.

Supplementary Fig. S4. Quantitative small-region intracellular calcium analysis shows evidence that $[Ca^{2+}]_i$ initiated at edge. $[Ca^{2+}]_i$ ratio in region ① (red curve) reaches to its 50% peak (green circle) earlier than in region ② (black circle on the blue curve).

Supplementary Fig. S5. The time course of temperature change in the Petri dish at different PL. Values are expressed as means ± std. (n = 3). AS: acoustic streaming.

Supplementary Fig. S6. Peak normalized intracellular calcium ratio change in P1TF cells when cultured in regular medium (red), regular medium with 3 μM thapsigargin (green), or in Ca^{2+}-free medium (brown) at various PLs (n ~ 6 for each case).

Supplementary Fig. S7. Temporal evolution of pressure (p_1) and streaming velocity (v_2) produced by the VD-SAW at 33 MHz. Dash lines indicate the time-averaged value of steady state obtained by using the frequency-domain solver.

Supplementary Fig. S8. Numerical simulation of the modified four-state Piezo1 gating model. (a) Illustrative diagram depicting the ‘‘first peak’’ (red), ‘‘last peak’’(blue), ‘‘tonic current’’ (orange) and current integral (area under the curve). (b) Experimental and simulated amplitudes of the ‘‘first peak’’ and ‘‘last peak’’ currents and ‘‘tonic’’ current as a function of stimulus frequency of a 4-s sinusoidal wave. (c) Experimental and simulated current integral during the last 2 s of a 4-s sinusoidal force stimulus as a function of stimulus frequency. Experimental measurements of membrane current[3] are expressed as means ± std. (n = 8-13).

Supplementary Fig. S9. Simulation of Piezo1 channel kinetics by the modified four-state gating model for PL = 0.01 and 0.1 ms. (a) & (b) The normalized shear stress (red), simulated Piezo1 open (blue), closed (magenta), inactivation 1 (black) and inactivation 2 (green) probabilities change in response to 60 s insonification for PL = 0.01 and 0.1 ms, respectively. (a1) & (b1) The magnified plot at the beginning of the insonification for PL = 0.01 and 0.1 ms, respectively. (a2) & (b2) The magnified plot toward the end of the insonification for PL = 0.01 and 0.1 ms, respectively.

SUPPLEMENTARY FIGURES

Supplementary Figure 1

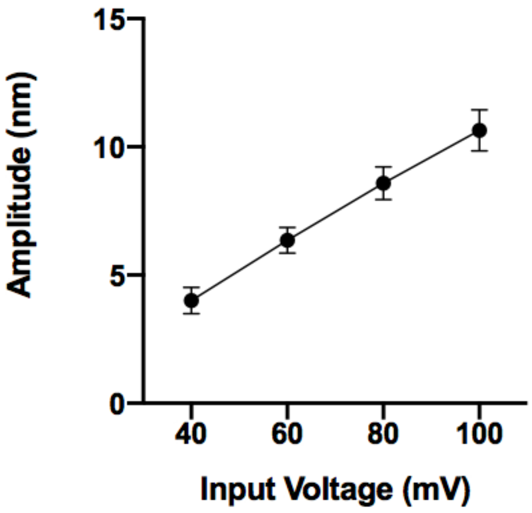

Supplementary Figure 2

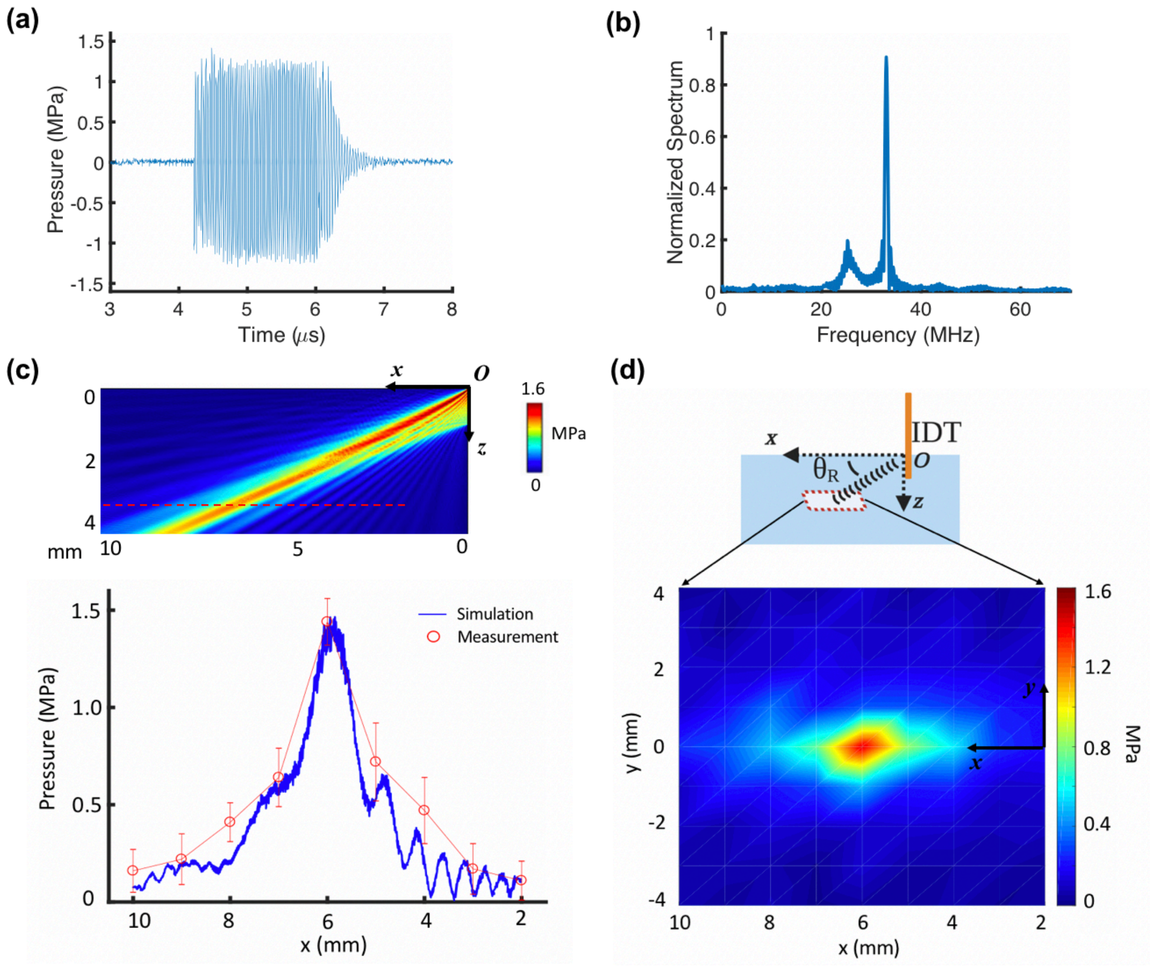

Supplementary Figure 3

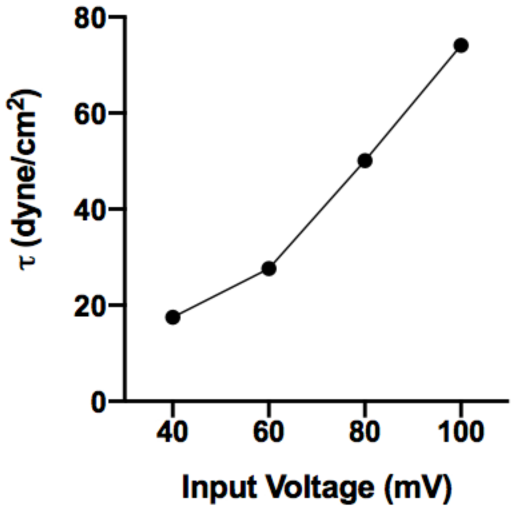

Supplementary Figure 4

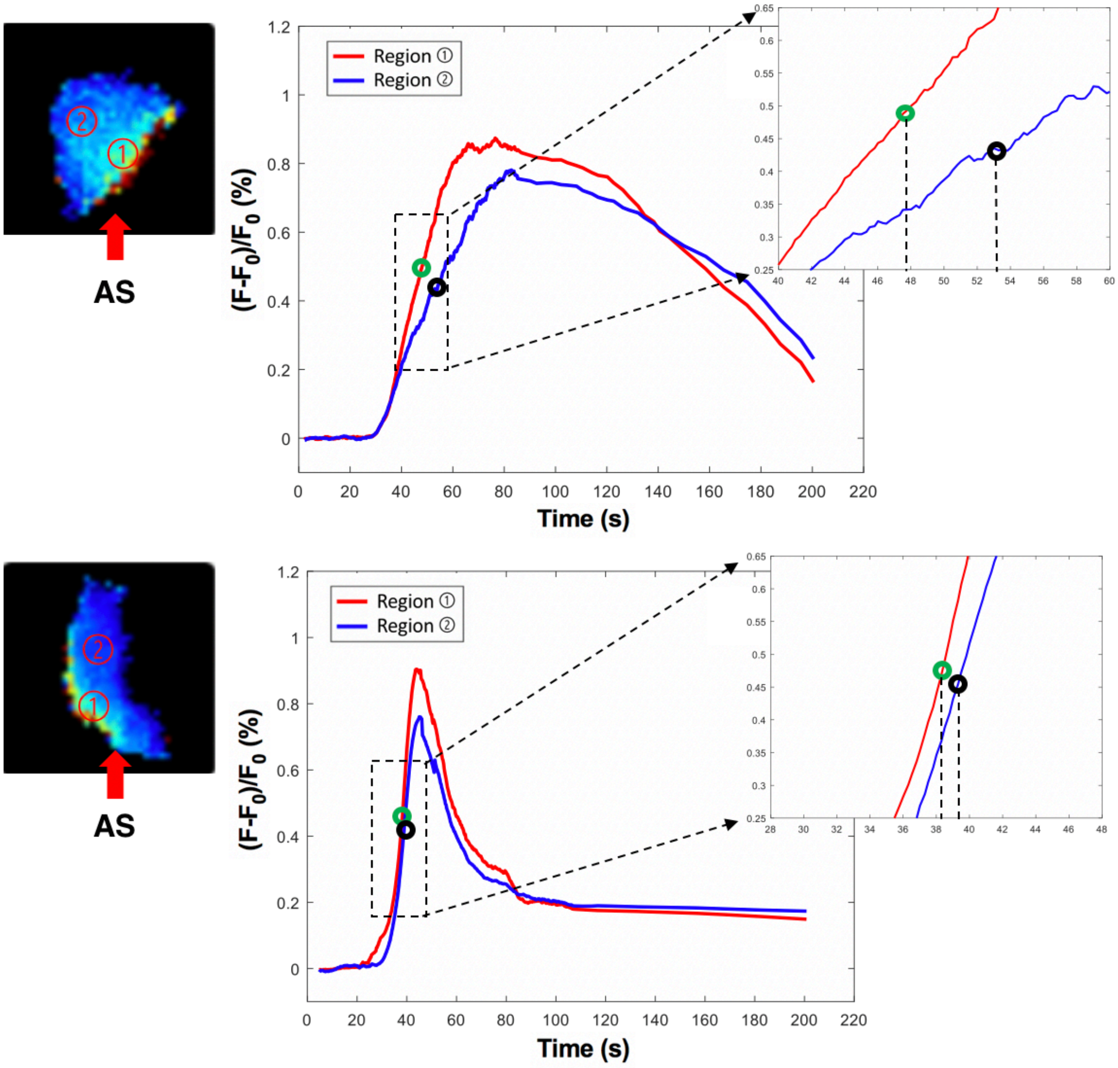

Supplementary Figure 5

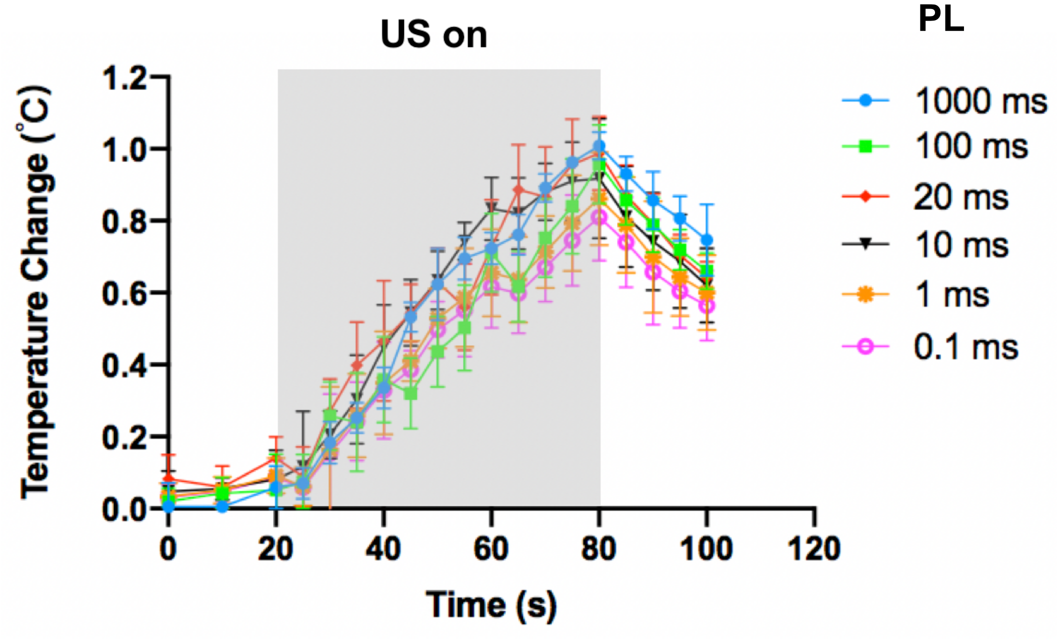

Supplementary Figure 6

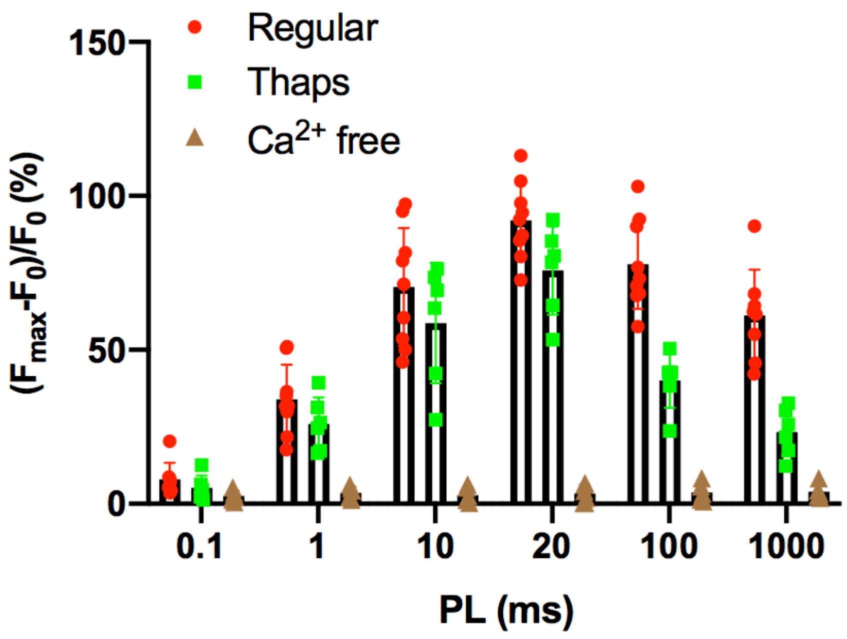

Supplementary Figure 7

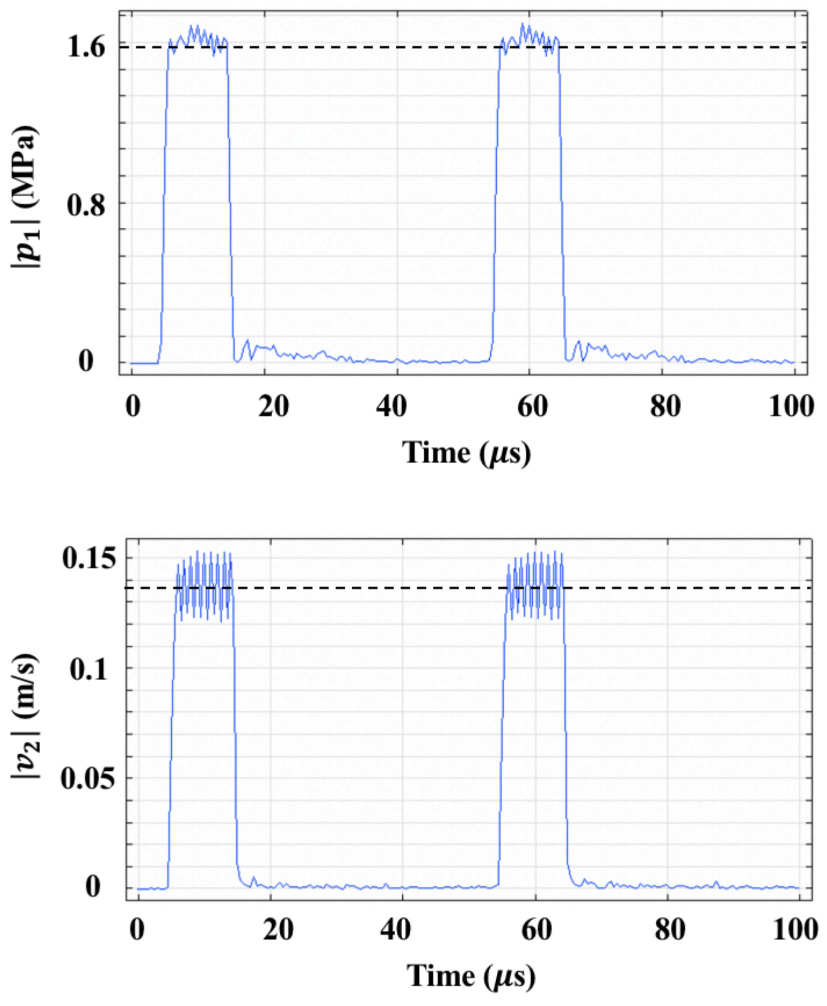

Supplementary Figure 8

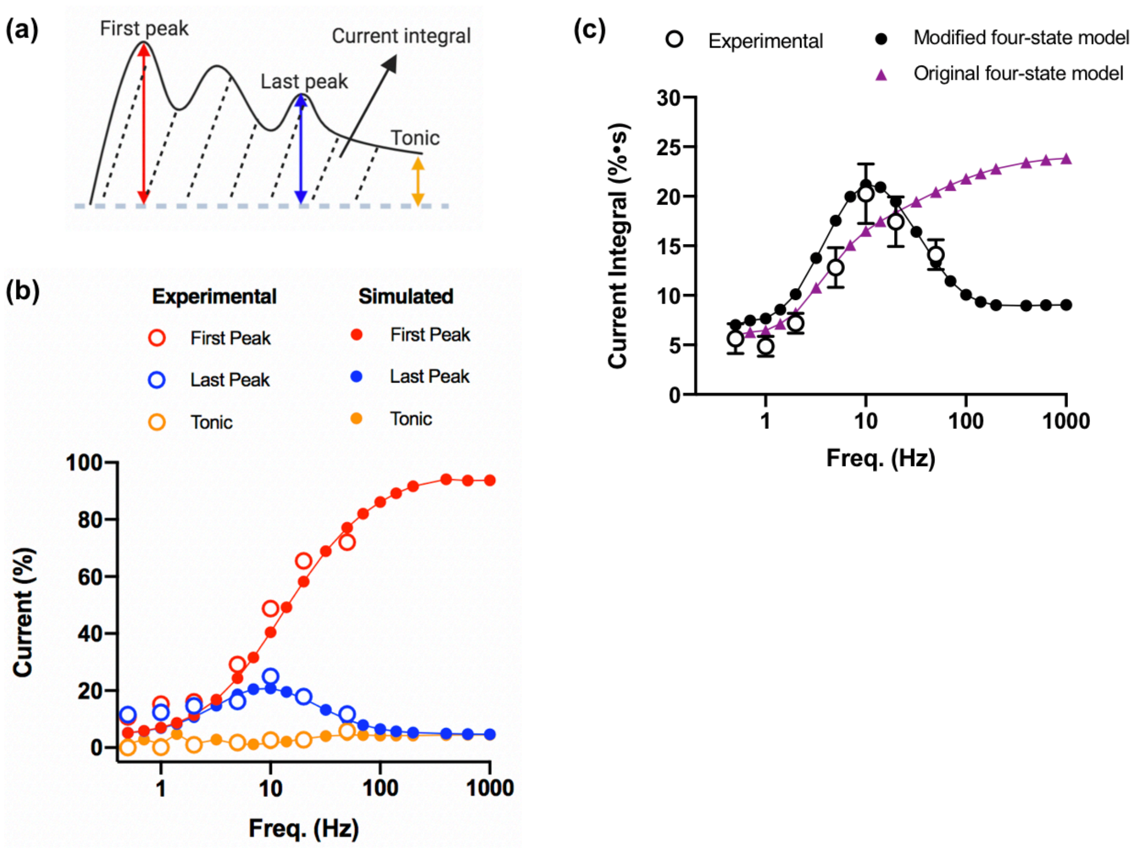

Supplementary Figure 9

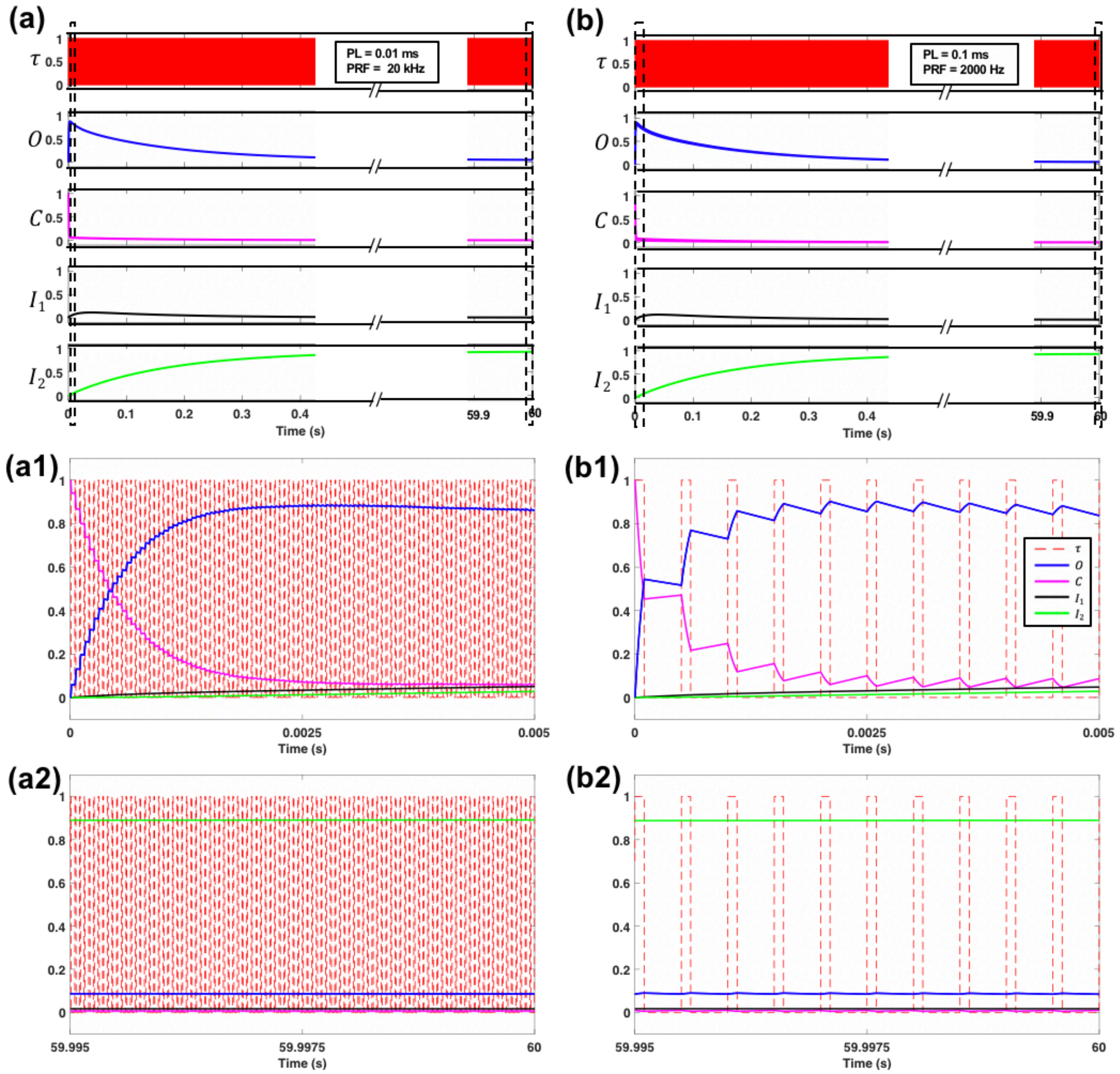

SUPPLEMENTARY REFERENCES LIST:

1 Guo, F. *et al.* Three-dimensional manipulation of single cells using surface acoustic waves. *Proc Natl Acad Sci U S A* **113**, 1522-1527, doi:10.1073/pnas.1524813113 (2016).

2 Muller, P. B. & Bruus, H. Theoretical study of time-dependent, ultrasound-induced acoustic streaming in microchannels. *Phys Rev E Stat Nonlin Soft Matter Phys* **92**, 063018, doi:10.1103/PhysRevE.92.063018 (2015).

3 Lewis, A. H., Cui, A. F., McDonald, M. F. & Grandl, J. Transduction of Repetitive Mechanical Stimuli by Piezo1 and Piezo2 Ion Channels. *Cell Rep* **19**, 2572-2585, doi:10.1016/j.celrep.2017.05.079 (2017).
